# Supplementary figures and images for: Tracing Genetic Exchange and Biogeography of Cryptococcus neoformans var. grubii at the Global Population Level
Source: Genetics. 2017 Jul 5;207(1):327–46. doi: 10.1534/genetics.117.203836 (PMC5586382; doi:10.1534/genetics.117.203836)

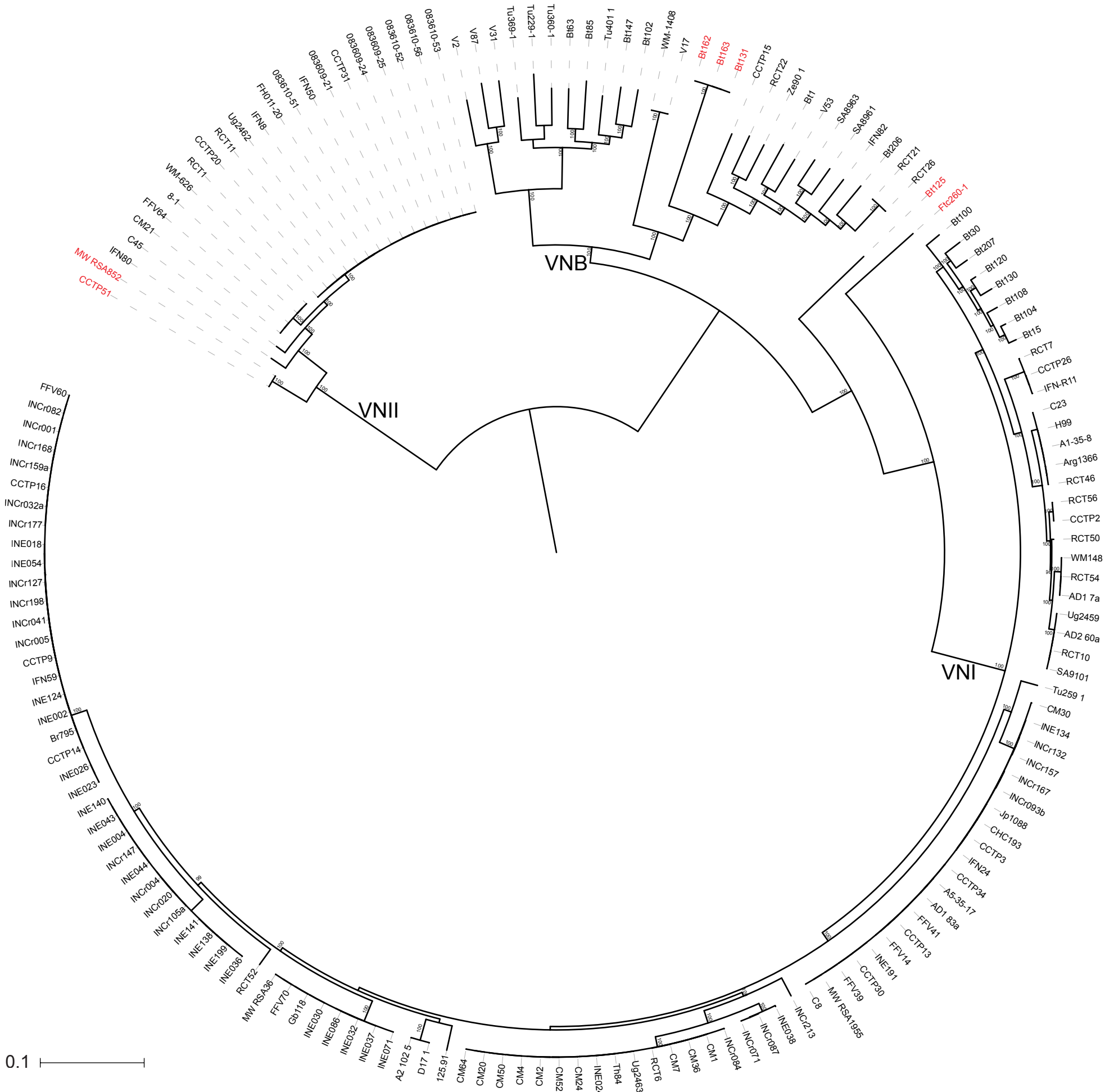

Tree scale: 0.1

Supplement: Supplementary file 1 [file 327FigureS1.pdf]

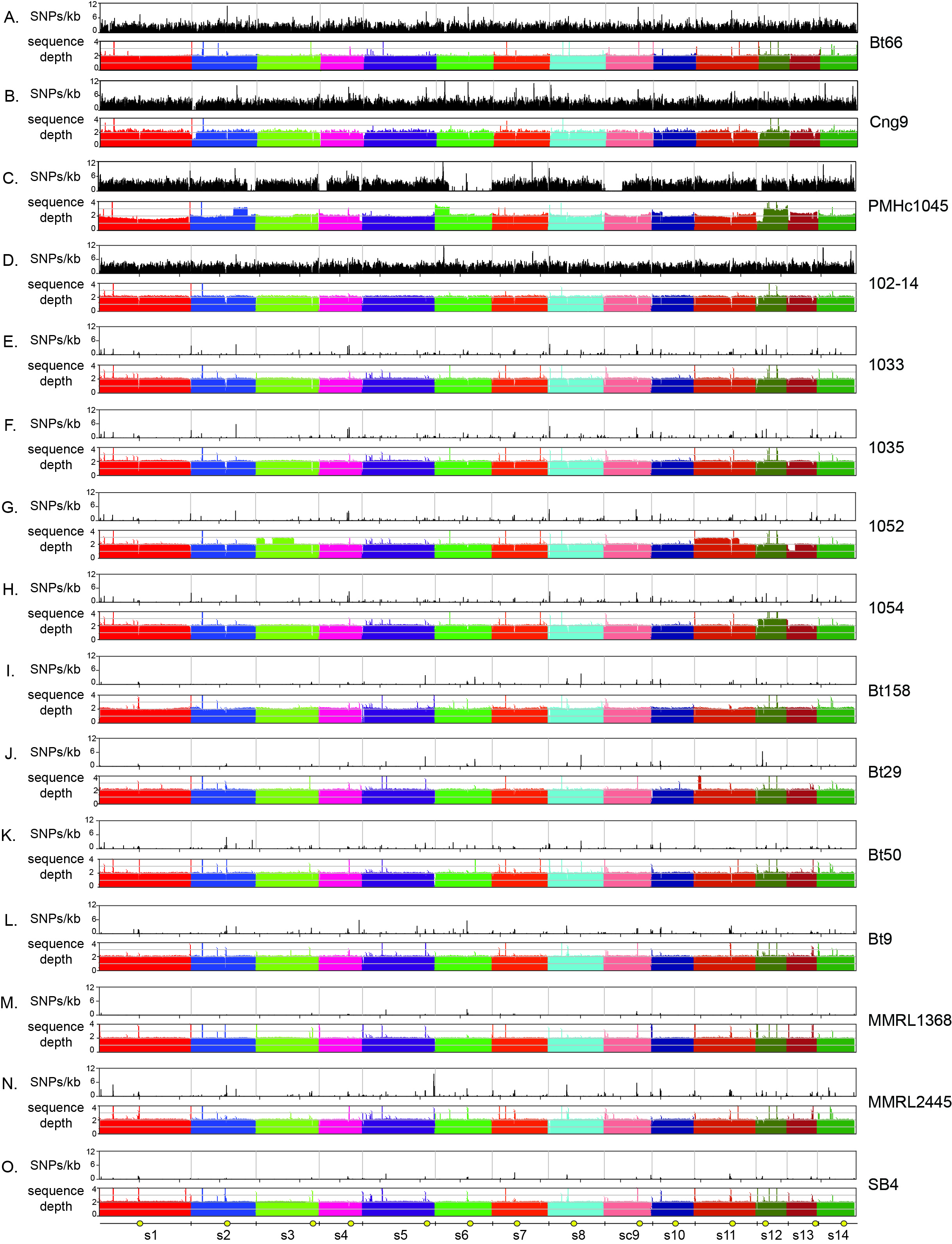

Supplement: Supplementary file 3 [file 327FigureS3.jpg]

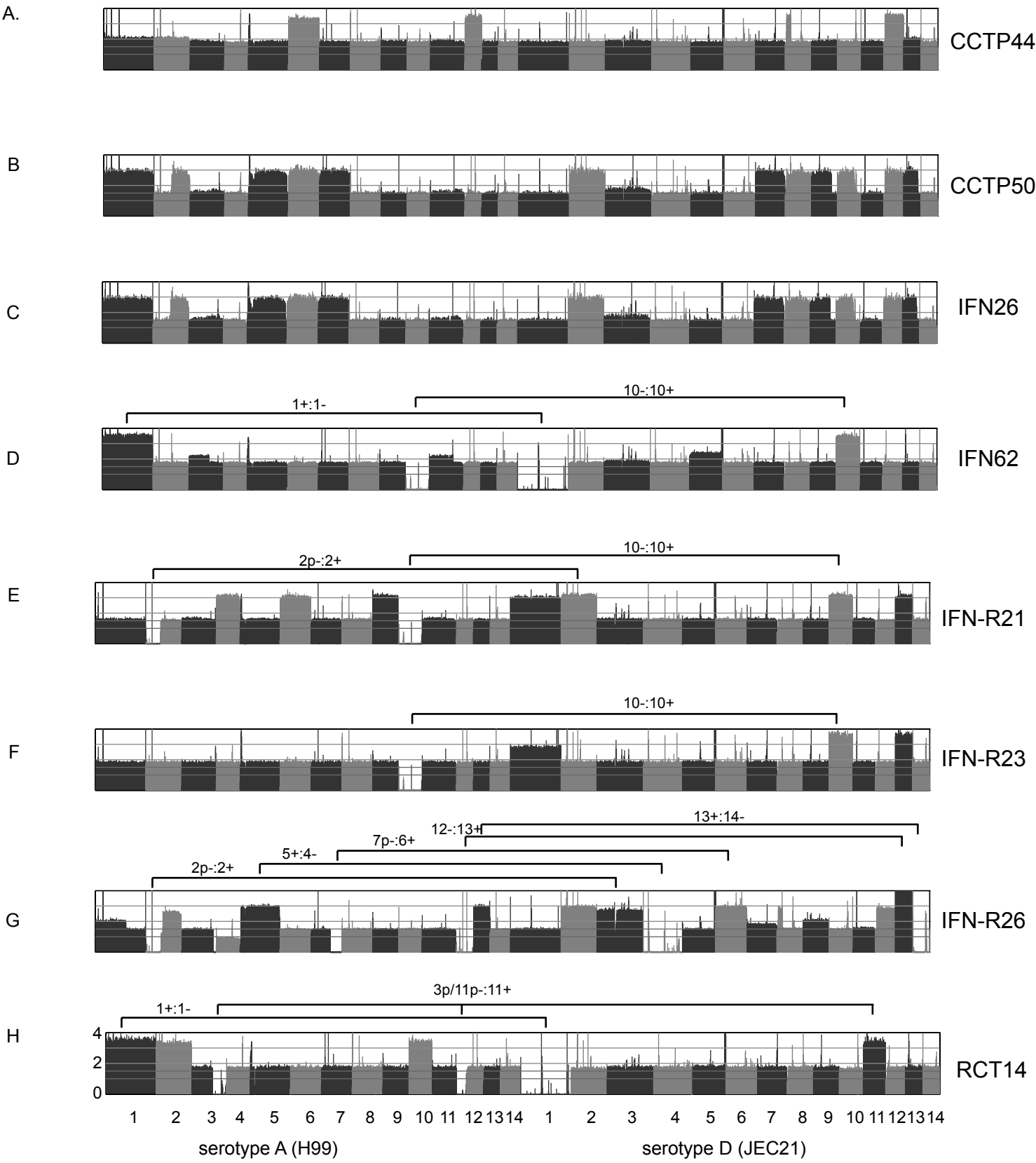

Supplement: Supplementary file 4 [file 327FigureS4.pdf]

A.

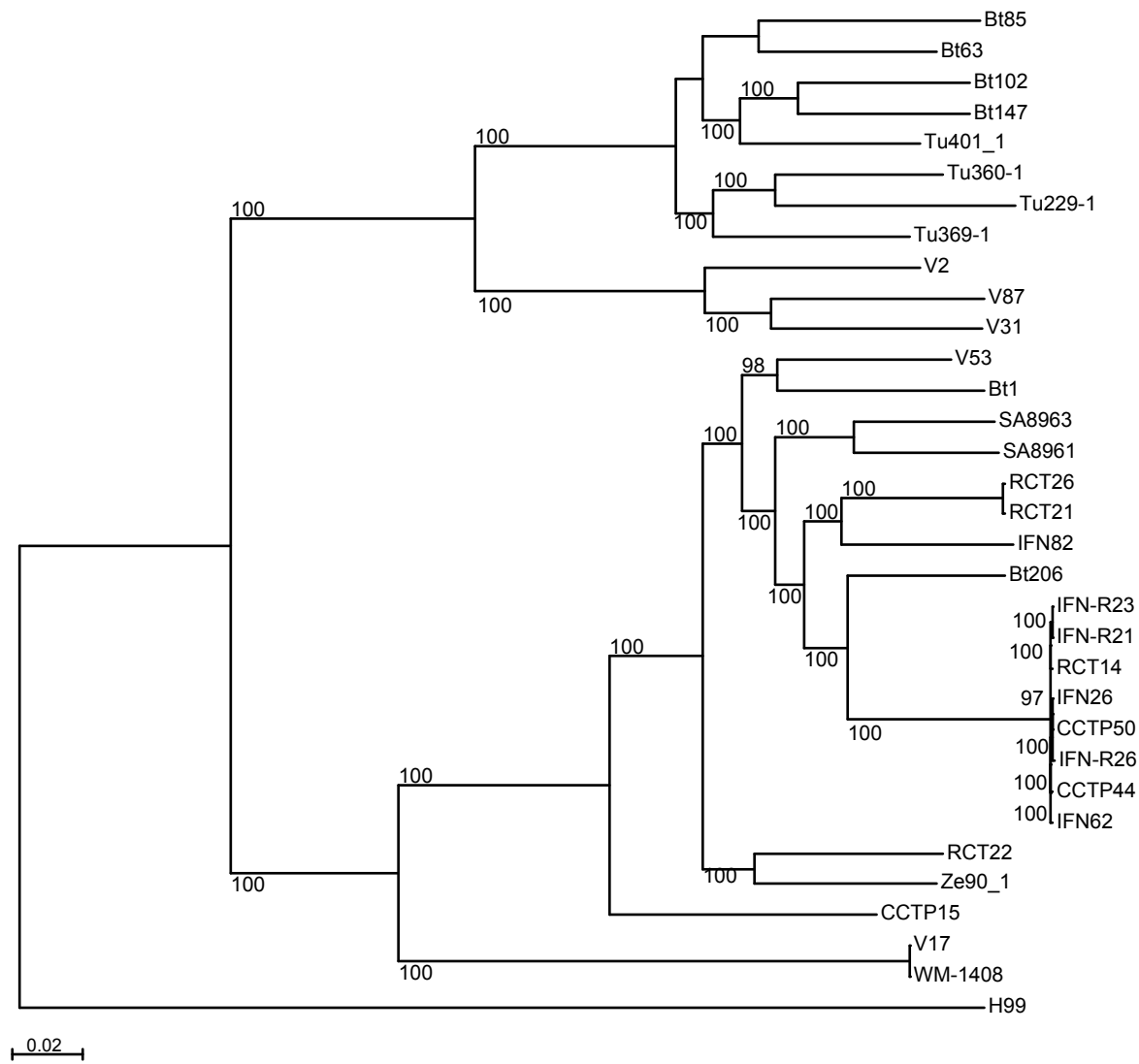

B.

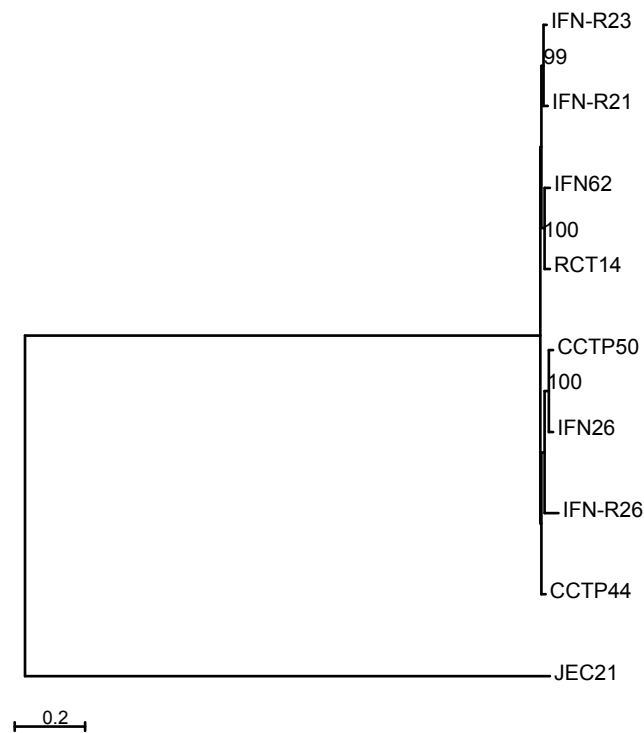

Supplement: Supplementary file 5 [file 327FigureS5.pdf]

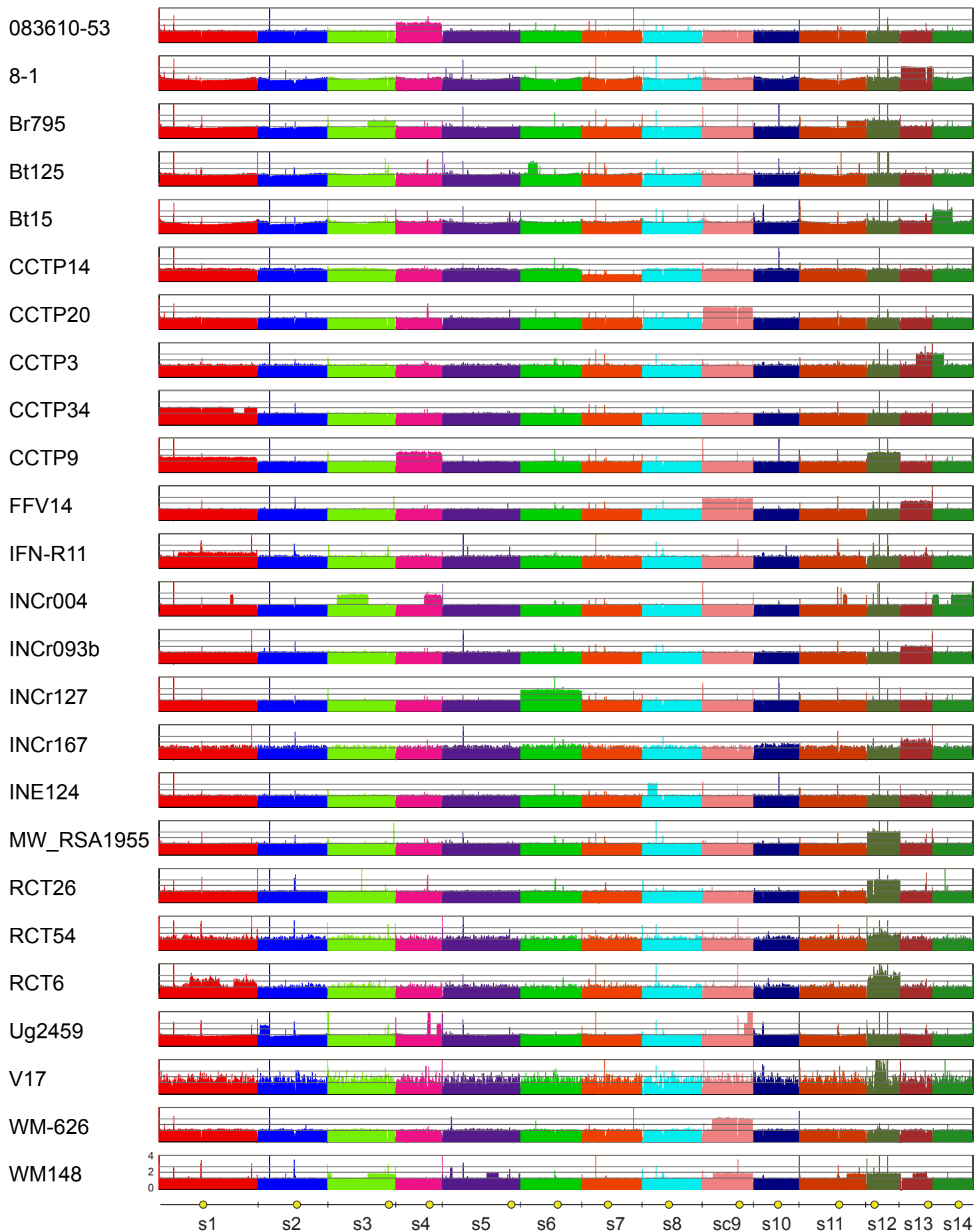

Supplement: Supplementary file 6 [file 327FigureS6.pdf]

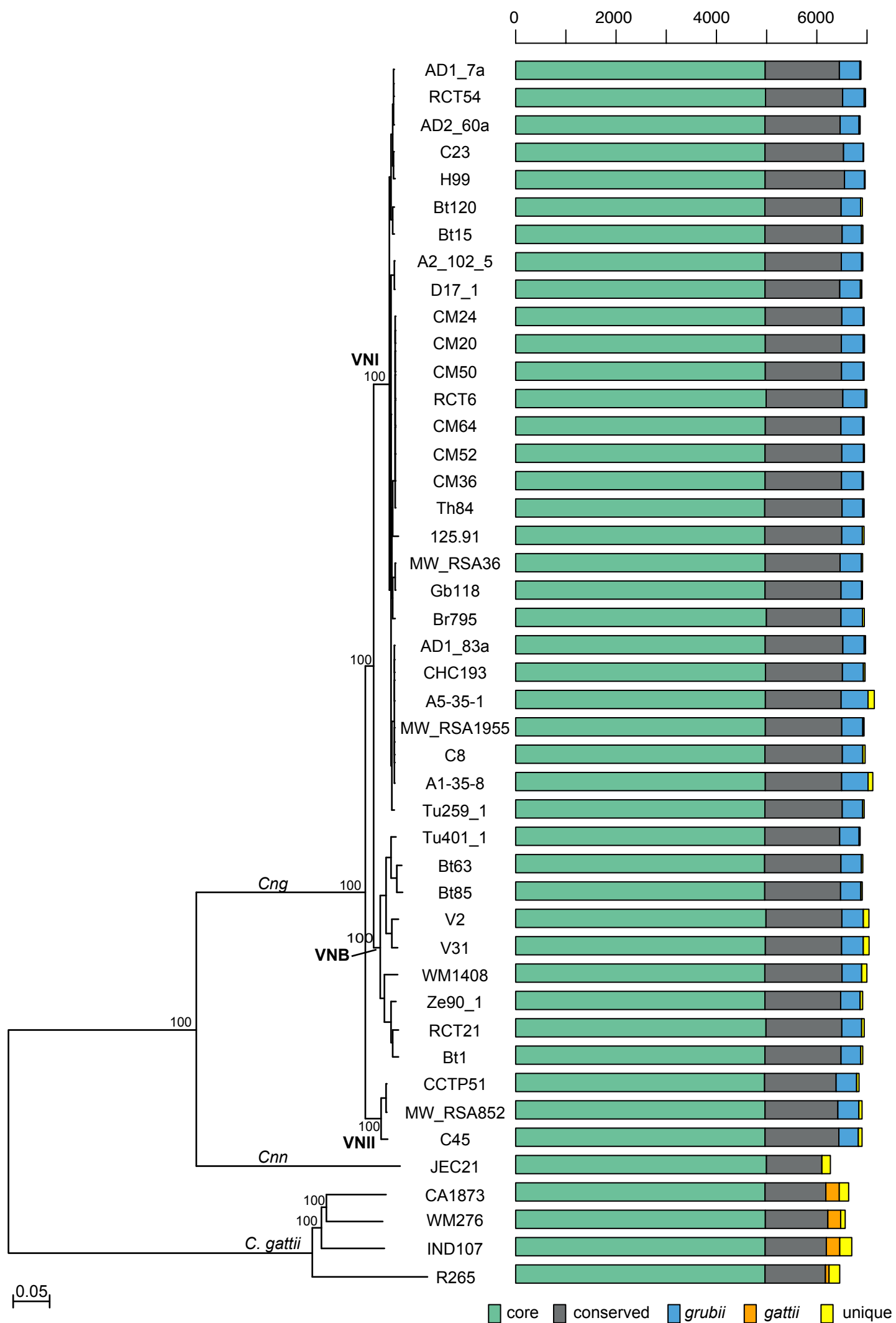

Supplement: Supplementary file 7 [file 327FigureS7.pdf]

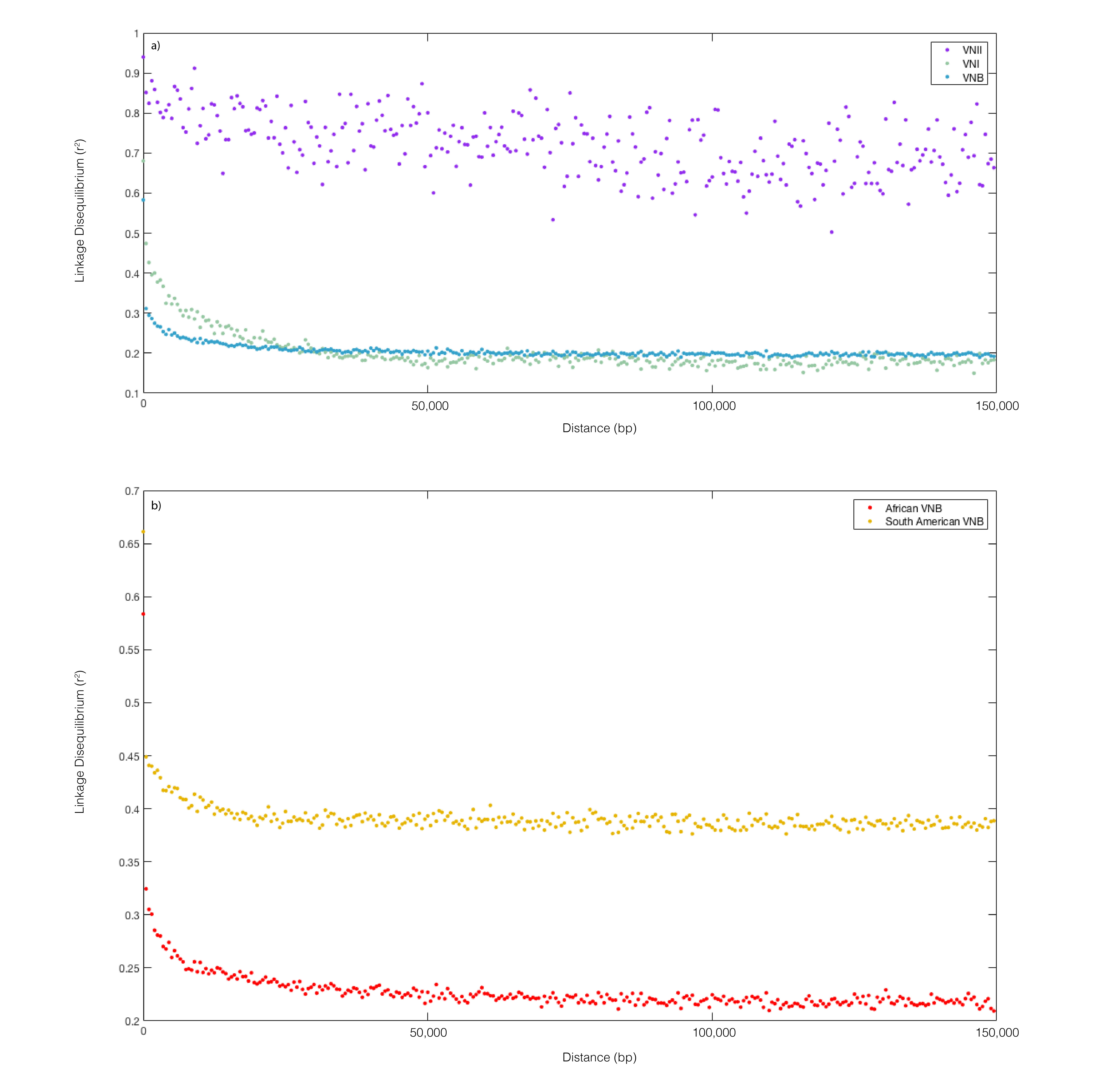

Supplement: Supplementary file 8 [file 327FigureS8.jpg]
