# Supplementary material for: Tracing Genetic Exchange and Biogeography of Cryptococcus neoformans var. grubii at the Global Population Level
Source: Genetics. 2017 Jul 5;207(1):327–46. doi: 10.1534/genetics.117.203836 (PMC5586382; doi:10.1534/genetics.117.203836)
Supplement: Supplementary file 2 [file 327FigureS2.pdf]

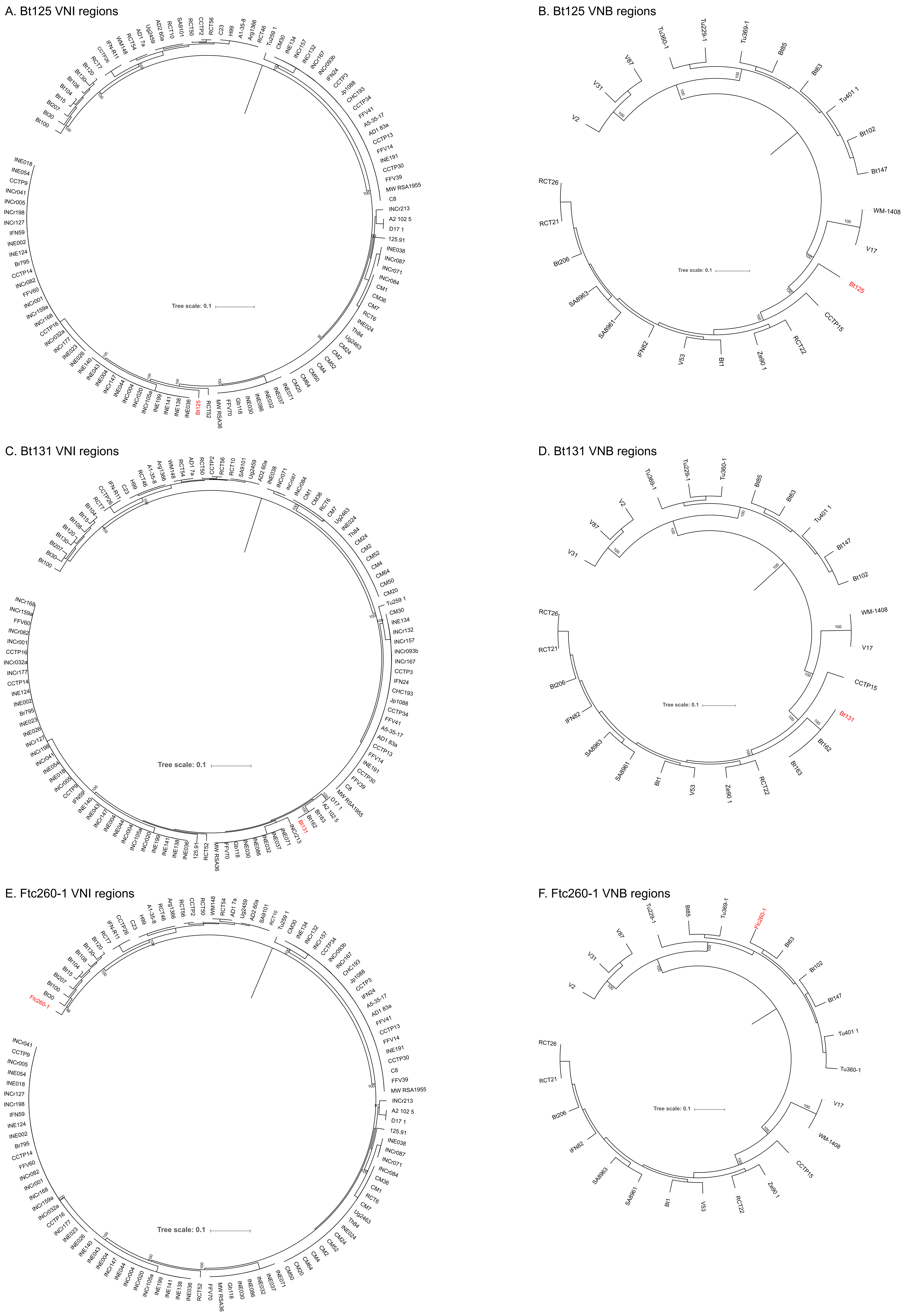
A. Bt125 VNI regions
Bt100
Bt30
Bt207
Bt15
Bt104
Bt108
Bt130
Bt120
RCT7
CCTP26
IFN-R11
WM148
RCT54
AD1 7a
Ug2459
AD2 60a
RCT10
SA9101
RCT50
CCTP2
RCT56
C23
H99
A1-35-8
Arg1366
RCT46
Tu259 1
CM30
INE134
INCr157
INCr132
INCr167
INCr093b
IFN24
CCTP3
Jp1088
CHC193
CCTP34
FFV41
A5-35-17
AD1 83a
CCTP13
FFV14
INE191
CCTP30
FFV39
MW RSA1955
C8
INCr213
A2 102 5
D17 1
125.91
INE038
INCr087
INCr071
INCr084
CM1
CM36
CM7
RCT6
INE024
Th84
Ug2463
CM24
CM2
CM52
CM4
CM50
CM64
CM20
INE071
INE037
INE032
INE086
INE030
Gb118
FFV70
MW RSA36
RCT52
Bt125
INE036
INE138
INE141
INE199
INCr105a
INCr020
INCr004
INE044
INCr147
INE004
INE043
INE140
INE026
INE023
INCr177
INCr032a
CCTP16
INCr168
INCr159a
INCr001
FFV60
INCr082
CCTP14
Br795
INE124
INE002
IFN59
INCr127
INCr198
INCr005
INCr041
CCTP9
INE054
INE018
Tree scale: 0.1
B. Bt125 VNB regions
V2
V31
V87
Tu360-1
Tu229-1
Tu369-1
Bt85
Bt63
Tu401 1
Bt102
Bt147
WM-1408
V17
Bt125
CCTP15
RCT22
Ze90 1
Bt1
V53
IFN82
SA8961
SA8963
Bt206
RCT21
RCT26
Tree scale: 0.1
C. Bt131 VNI regions
D. Bt131 VNB regions
Bt131
Bt162
Bt163
Tree scale: 0.1
E. Ftc260-1 VNI regions
Ftc260-1
Tree scale: 0.1
F. Ftc260-1 VNB regions
Ftc260-1
Tree scale: 0.1
