# Supplementary material for: Tracing Genetic Exchange and Biogeography of Cryptococcus neoformans var. grubii at the Global Population Level
Source: Genetics. 2017 Jul 5;207(1):327–46. doi: 10.1534/genetics.117.203836 (PMC5586382; doi:10.1534/genetics.117.203836)
Supplement: Supplementary file 9 [file 327FileS1.docx]

**File S1:**

**Gene conservation in *C. neoformans***

By examining gene conservation to *C. gattii* and C. *neoformans* var. *neoformans*, we identified a total of 239 gene clusters specific to *C. neoformans* var. *grubii* and present in at least 36 of the 40 genomes (90% or more). While the 181 of these clusters encode hypothetical proteins with no predicted function, the remaining 59 include a highly diverse set of functional predictions (**Table S6**). Of these 59, 8 encode for transporters, including the inositol transporter *ITR1A*. Other than ITR1A, only two other named genes (Inglis *et al.* 2014) are this set of 58. *KRE63*, conserved in 39 of the 40 genomes, is related to Beta-glucan synthase genes but deletion did not detect any phenotypic changes (Gilbert *et al.* 2010). *HAP5*, a histone-like transcription factor, is also conserved in 39 of 40 genomes; while this sequence is found in other species, the gene structure in C. *neoformans* var. *neoformans* and *C. gattii* is fused to that of the adjacent gene in *C. neoformans* var. *grubii*.

**Loss-of-Function Mutations in *C. neoformans* var. *grubii* Lineages**

To identify loss-of-function mutations in read data, we used the SNPs and indels predicted as described in Methods. We then annotated the variants using SnpEff (Cingolani *et al.* 2012). For VNB and VNII, we identified genes where all members of each lineage were predicted to have loss-of-function mutations. As the reference genome is in VNI, for VNI loss-of-function mutations we identified genes that had extended stop codons in both VNB and VNII. For each gene with a known function (Brown *et al.* 2014), we evaluated the effect of the mutation on the amino acid sequence. In all cases, genes had either 1) a compensatory frameshift within a few bases reverting the effect, 2) a compensatory SNP at an adjacent position preventing a premature stop codon from being created, or 3) the frameshift only altered the first or last 30 or fewer amino acids of the protein. Based on this, we found no convincing evidence for loss-of-function any genes with known functions in any of the three lineages.

**Supplemental References**

Brown, J. C. S., J. Nelson, B. VanderSluis, R. Deshpande, A. Butts *et al.*, 2014 Unraveling the biology of a fungal meningitis pathogen using chemical genetics. Cell 159: 1168–1187.

Cingolani, P., A. Platts, L. L. Wang, M. Coon, T. Nguyen *et al.*, 2012 A program for annotating and predicting the effects of single nucleotide polymorphisms, SnpEff: SNPs in the genome of Drosophila melanogaster strain w1118; iso-2; iso-3. Fly (Austin) 6: 80–92.

Gilbert, N. M., M. J. Donlin, K. J. Gerik, C. A. Specht, J. T. Djordjevic *et al.*, 2010 KRE genes are required for β-1,6-glucan synthesis, maintenance of capsule architecture and cell wall protein anchoring in *Cryptococcus neoformans*. Mol. Microbiol. 76: 517–534.

Inglis, D. O., M. S. Skrzypek, E. Liaw, V. Moktali, G. Sherlock *et al.*, 2014 Literature-Based Gene Curation and Proposed Genetic Nomenclature for *Cryptococcus*. Eukaryot. Cell 13: 878–883.
